# Mentoring at times of crises: Personal reflections on mentoring relationships during COVID‐19

**DOI:** 10.1111/eje.12804

**Published:** 2022-05-06

**Authors:** Hoda S. Wassif, Charlotte Wake

**Affiliations:** ^1^ Faculty of Health and Social Sciences University of Bedfordshire Luton UK

**Keywords:** Covid19, dental, education, mentoring, support

## Abstract

COVID‐19 presented a huge challenge for practice, education and all interactions, and mentorship was no different. The purpose of this commentary is to reflect on the juxtaposition between mentors and mentees in dental education during COVID‐19. This commentary will focus on the interaction between mentor/mentee outside clinical practice and in relation to supporting and mentoring dental practitioners in the context of postgraduate education. The aim is to share our learning from this experience with other dental educators beyond COVID‐19.

## 1 INTRODUCTION

Mentorship^1^ is one of the professional interactions that can be truly noticed when it is not there or when it is done badly. At times, mentorship can be seen as an “added extra” or simply a paperwork exercise. It does not help when mentoring is conflicted with supervision, management and/or training. COVID‐19 presented the added challenges of stress, pressure and isolation^2^ for many. This commentary is a look‐back at mentorship during the time of COVID‐19 and how we can learn from this unique time to strengthen our approach to professional mentoring in dental education.

The overlap between mentoring, supervision and training can make mentoring seem to be a pathway with milestones in mind rather than a process to support, guide and “be with” the mentee to find their own voice with mentors running the risk of focusing on supervision and managing performance whilst true mentorship is lost amongst predetermined milestones^3^.

As both mentors and mentees battled in dealing with the challenges presented by the pandemic, the interconnectivity of both mentor and mentee, and the circular nature of mentorship^4^ were under sharper focus. The ongoing interaction of the mentorship process was dynamic and beneficial to both parties rather than the traditional dependence of the mentee on their mentor and the perception that the mentor has all the answers. This was a unique time where both mentors and mentees were experiencing the same challenge, similar pressures and no predetermined pathway to travel. Additionally, COVID‐19 presented a sense of urgency to personal development^5^ with more reflection on what matters and the value of professional goals. At the peak of the pandemic, more considerations were made to the added benefits of mentoring^4^ which was shared by mentors and mentees alike. It was easy to share ideas, listen to mentee's experience and rejoice in their successes. Regular mentoring meeting was a needed reminder about the fulfilment of being a dental educator and the pride of making a difference. As smaller connections were lost due to the pandemic, much attention was needed with “informal” catch‐up with mentees. Using social media helped with that small informal connection outside the planned, formal mentoring time.

Mentorship was considered as a holistic activity to care about the mentee, their progress and their well‐being. The interaction helped to put the mentee's agenda at the centre of discussion and offered a point of focus in the middle of the uncertainty of this global challenge. The mentorship platform ensured that small steps were celebrated and that well‐being and resilience were part of the ongoing discussion.

For mentees, mentorship became a real foundation for personal growth, and when loneliness and uncertainty were part of the pandemic challenge, having a mentor gave this difficult time an anchorage and stability with some added support both professionally and personally.

Within dental education, mentoring dynamics^6^ may come naturally to many. However, during the pandemic, both mentors and mentees were experiencing the same event at the same time with the same constraints on their development. The pandemic focused on the interaction not only to support the mentees but also to understand how mentors were dealing with the same challenge at the same time. This mentorship worked effectively with trust and openness as key skills^7^ to create an environment that was conducive to reflection for the benefits of both mentors and mentees.

## 2 FINAL THOUGHTS

This reflection came as a result of different discussions about developing as a dental educator and how easy (to a degree) it is to help and support the development of clinical skills and how challenging it is to develop reflection, insight and resilience. During the pandemic, there was a unique point where all had to pause, take stock and reconsider. Having invested in a mentor/mentee relationship prior to the pandemic where trust and rapport were already established, it was natural to carry on with mentorship. This was an opportunity for the mentor to lead by example at times of crises, and for the mentee, it was an invaluable chance to offer a different perspective about their future development.

The pandemic offered a reminder to refocus on the mentee's agenda and their own personal goals and not to be consumed by predetermined expectations. It also highlighted the role of the mentor not to offer ready‐made answers but to support mentees to explore their own approaches and their own answers. Investing in holistic mentorship in dental education can be rewarding for both mentors and mentees and can help in developing their roles for their benefit and the benefit of the profession.

## CONFLICT OF INTEREST

We do not believe there is a conflict of interest to disclose.

## Data Availability

Data sharing not applicable to this article as no datasets were generated or analysed during the current study.
